# Supplementary material for: Csf1r-mediated depletion of myeloid cells prevents dopaminergic neuron loss during chronic colitis
Source: J Neuroinflammation. 2026 Jun 27;23:214. doi: 10.1186/s12974-026-03926-9 (PMC13309958; doi:10.1186/s12974-026-03926-9)
Supplement: Supplementary file 1 — Supplementary Material 1. [file 12974_2026_3926_MOESM1_ESM.docx]

Supplementary file of figures and tables


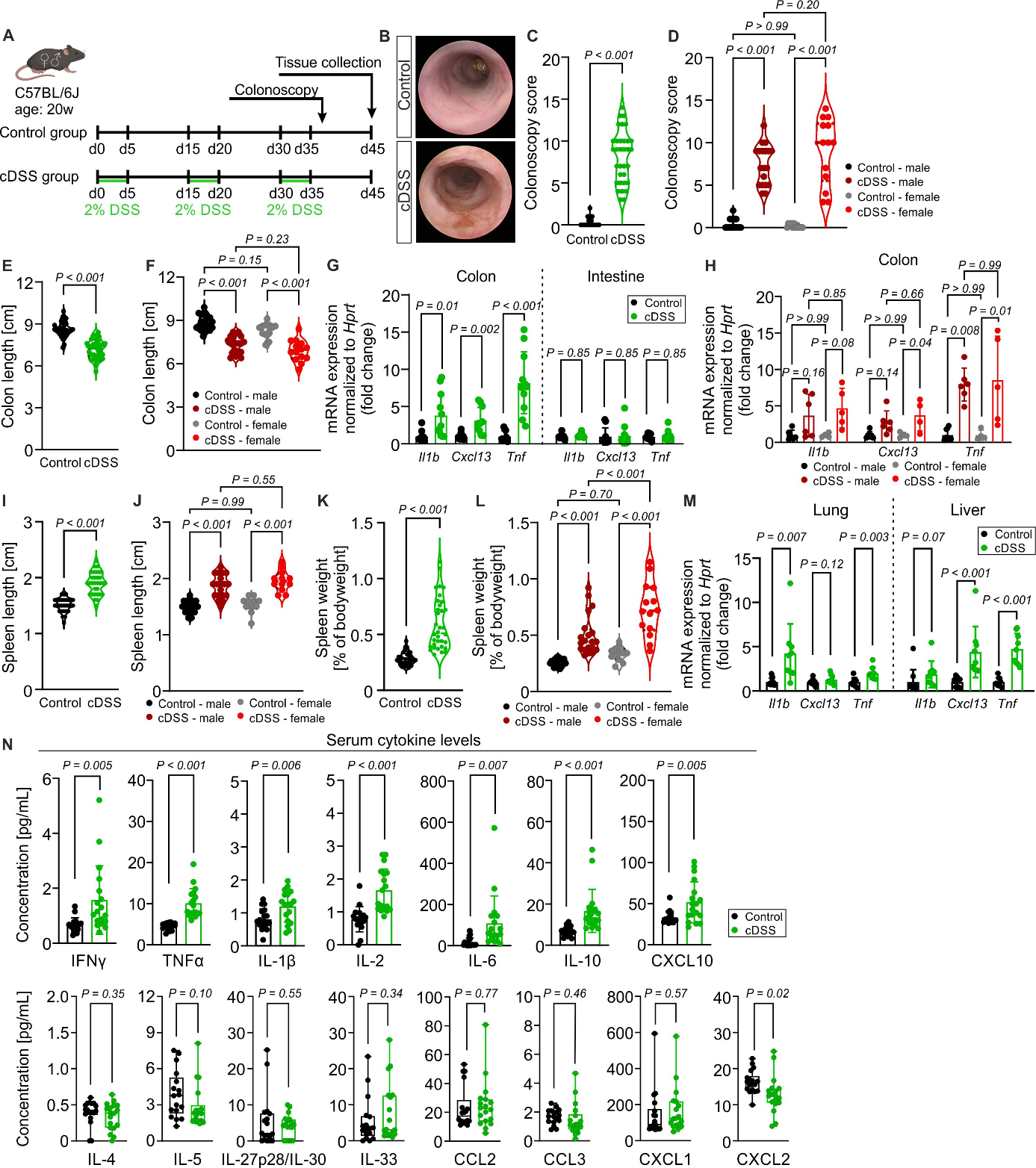


**Figure S1. Cyclic DSS treatment induces chronic colitis and systemic inflammation in C57BL/6J mice. A** Timeline of colitis induction in male and female C57BL/6J mice aged 20 weeks at the start of the experiment. **B** Representative colonoscopy images. **C** Colonoscopy scores, mixed sex (*n* = 32-33 mice per group; pooled of four independent experiments). **D** Colonoscopy scores, split by sex (*n* = 12-20 mice per group; Control: 20 male, 12 female, cDSS: 19 male, 14 female; pooled of four independent experiments; Two-way ANOVA with Tukey’s multiple comparisons test). **E** Colon length post dissection, mixed sex (*n* = 32-34 mice per group; pooled of four independent experiments). **F** Colon length post dissection, split by sex (*n* = 12-20 mice per group; Control: 20 male, 12 female, cDSS: 20 male, 14 female; pooled of four independent experiments; Two-way ANOVA with Tukey’s multiple comparisons test). **G** Expression of the inflammation-associated genes *Il1b*, *Cxcl13*, and *Tnf* in the colon and small intestine, mixed sex (*n* = 10-11 mice per group; multiple unpaired *t*-tests). **H** Expression of the inflammation-associated genes *Il1b*, *Cxcl13*, and *Tnf* in the colon and small intestine, split by sex (*n* = 4-6 mice per group; Control: 6 male, 4 female, cDSS: 6 male, 5 female; Two-way ANOVA with Tukey’s multiple comparisons test). **I** Spleen length, mixed sex (*n* = 29-31 mice per group; pooled of four independent experiments). **J** Spleen length, split by sex (*n* = 12-20 mice per group; Control: 18 male, 11 female, cDSS: 19 male, 12 female; pooled of four independent experiments; Two-way ANOVA with Tukey’s multiple comparisons test). **K** Spleen weight in percentage of bodyweight, mixed sex (*n* = 32-34 mice per group; pooled of four independent experiments). **L** Spleen weight in percentage of bodyweight, split by sex (*n* = 12-20 mice per group; Control: 20 male, 12 female, cDSS: 20 male, 14 female; pooled of four independent experiments; Two-way ANOVA with Tukey’s multiple comparisons test). **M** Expression of the inflammation-associated genes *Il1b*, *Cxcl13*, and *Tnf* in the lung and liver, mixed sex (*n* = 9-10 mice per group; Control: 6 male, 4 female, cDSS: 5 male, 4 female; multiple unpaired *t*-tests). **N** Serum concentrations of the inflammation-associated cytokines IFNγ, TNFα, IL-1β, IL-2, IL-6, IL-10, CXCL10, IL-4, IL-5, IL-27p28/IL-30, IL-33, CCL2, CCL3, CXCL1 and CXCL2 (*n* = 18 mice per group; Control: 14 male, 4 female, cDSS: 14 male, 4 female;). Two-tailed, unpaired *t*-test, if not otherwise indicated. Each point represents the value of one mouse. Data are presented as mean ± s.d. Icons in **A** were created with BioRender.com.


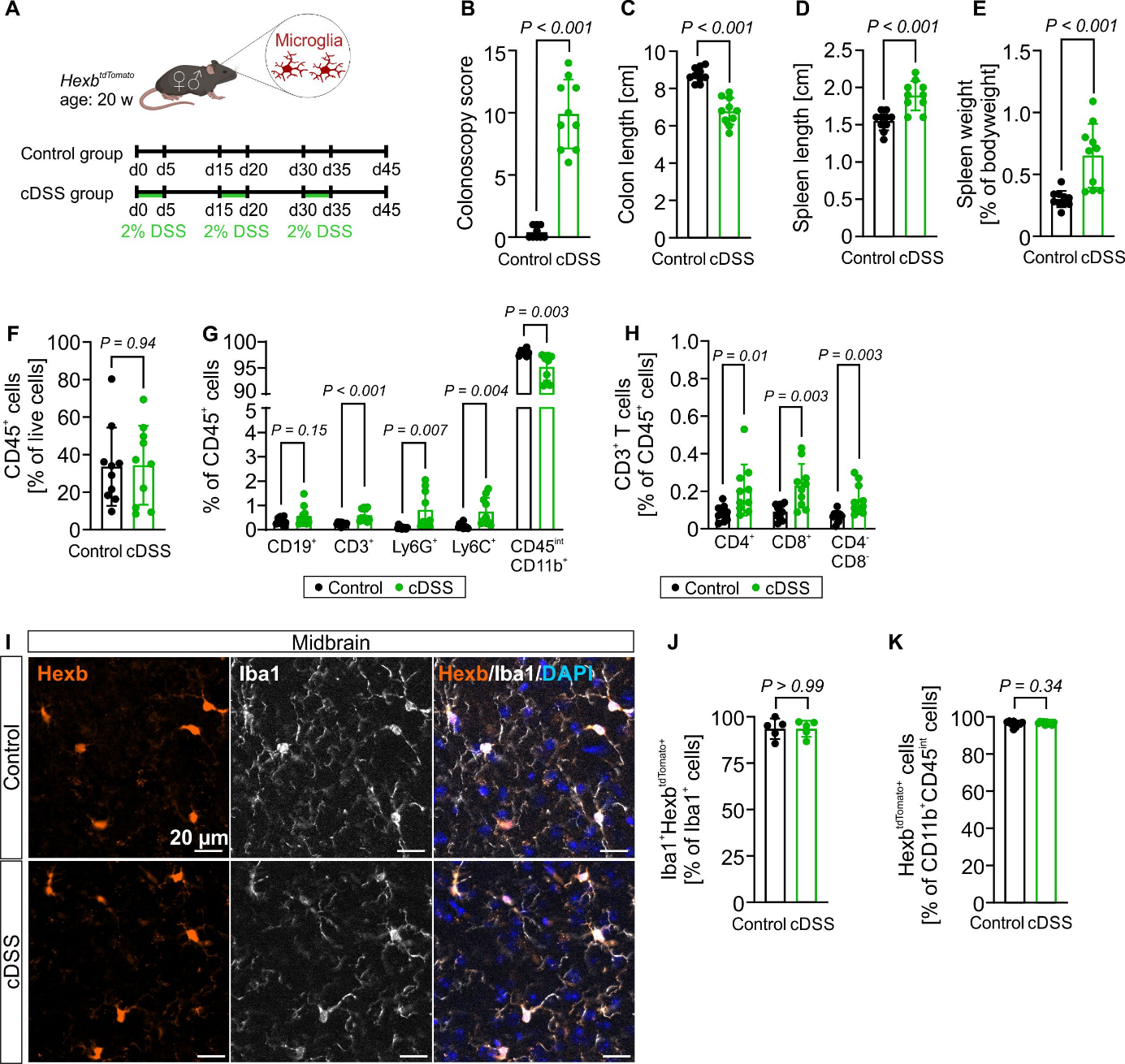


**Figure S2. Cyclic DSS treatment induces chronic colitis and immune cell response in the whole brain of *Hexb^tdTomato^* mice while microglial Hexb^tdTomato^ expression is maintained. A** Timeline of colitis induction in male and female *Hexb^tdTomato^* mice aged 20 weeks at the start of the experiment. **B** Colonoscopy score on day 36. **C** Colon length post dissection. **D** Spleen length post dissection. **E** Spleen weight in percentage of bodyweight. **F**, **G**, **H** Flow cytometry analysis of CD45^+^ immune cells (**F**), CD19^+^ B cells, CD3^+^ T cells, Ly6G^+^ neutrophils, Ly6C^+^ monocytes, and CD45^int^CD11b^+^ microglia as percentage of CD45^+^ cells (**G**), and CD3^+^CD4^+^, CD3^+^CD8^+^, and CD3^+^CD4^-^CD8^-^ T cells as percentage of CD45^+^ cells (**H**) in whole brain tissue. **I**, Immunostaining for Iba1 (white) and Hexb (tdTomato) in brain tissue of *Hexb^tdTomato^* mice. Scale bar, 20 µm. **J**, Quantification of Iba1^+^Hexb^tdTomato+^ cells in percentage of Iba1^+^ cells in the midbrain. **K**, Flow cytometry analysis of Hexb^tdTomato+^ cells in percentage of CD11b^+^CD45^int^ cells. The results are from *n* = 10 (Control: 5 male, 5 female, cDSS: 4 male, 6 female; **B - H**, **K**) and *n* = 5 (Control: 2 male, 3 female, cDSS: 1 male, 4 female; **J**) mice per group. Two-tailed, unpaired *t*-test, if not otherwise indicated. Each point represents the value of one mouse. Data are presented as mean ± s.d. Icons in **A** were created with BioRender.com.


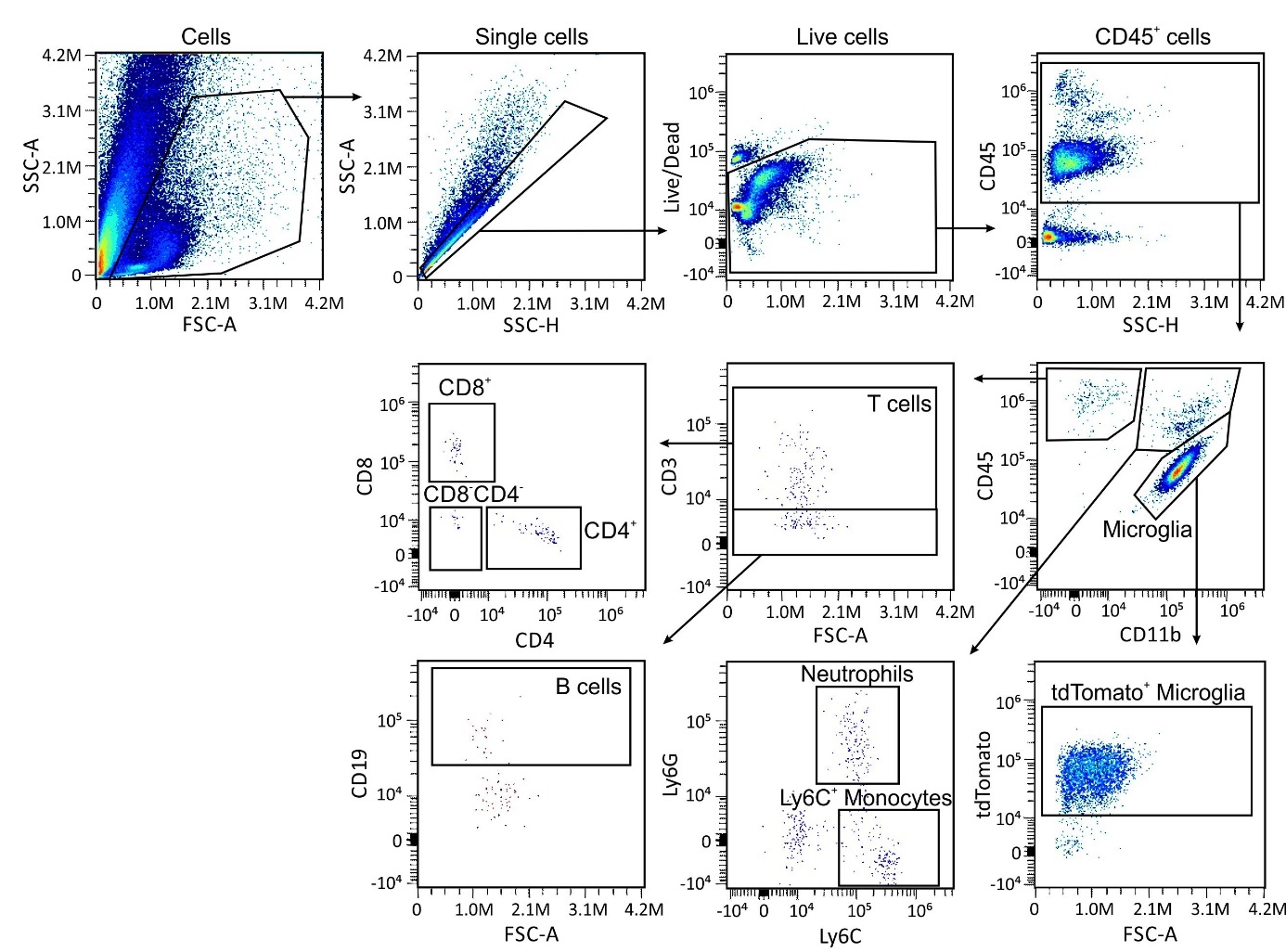


**Figure S3. Flow cytometry gating strategy of brain immune cells isolated from cDSS-treated and control *Hexb^tdTomato^* mice.** Cells were gated on SSC-A and SSC-H to remove doublets. Singlets negative for the Live/Dead fixable Aqua stain were considered as live cells. CD45^+^ live cells were gated on CD11b to identify CD11b^+^CD45^int^ microglia that were further gated on tdTomato to determine tdTomato^+^ microglia. CD11b^+^CD45^+^ cells were gated on Ly6G and Ly6C to distinguish Ly6G^+^ neutrophils from Ly6C^+^ monocytes. CD11b^-^CD45^+^ cells were gated on CD3 (T cells) and CD19 (B cells), and CD3^+^ cells were further gated on CD8 and CD4 to identify T cell subpopulations.


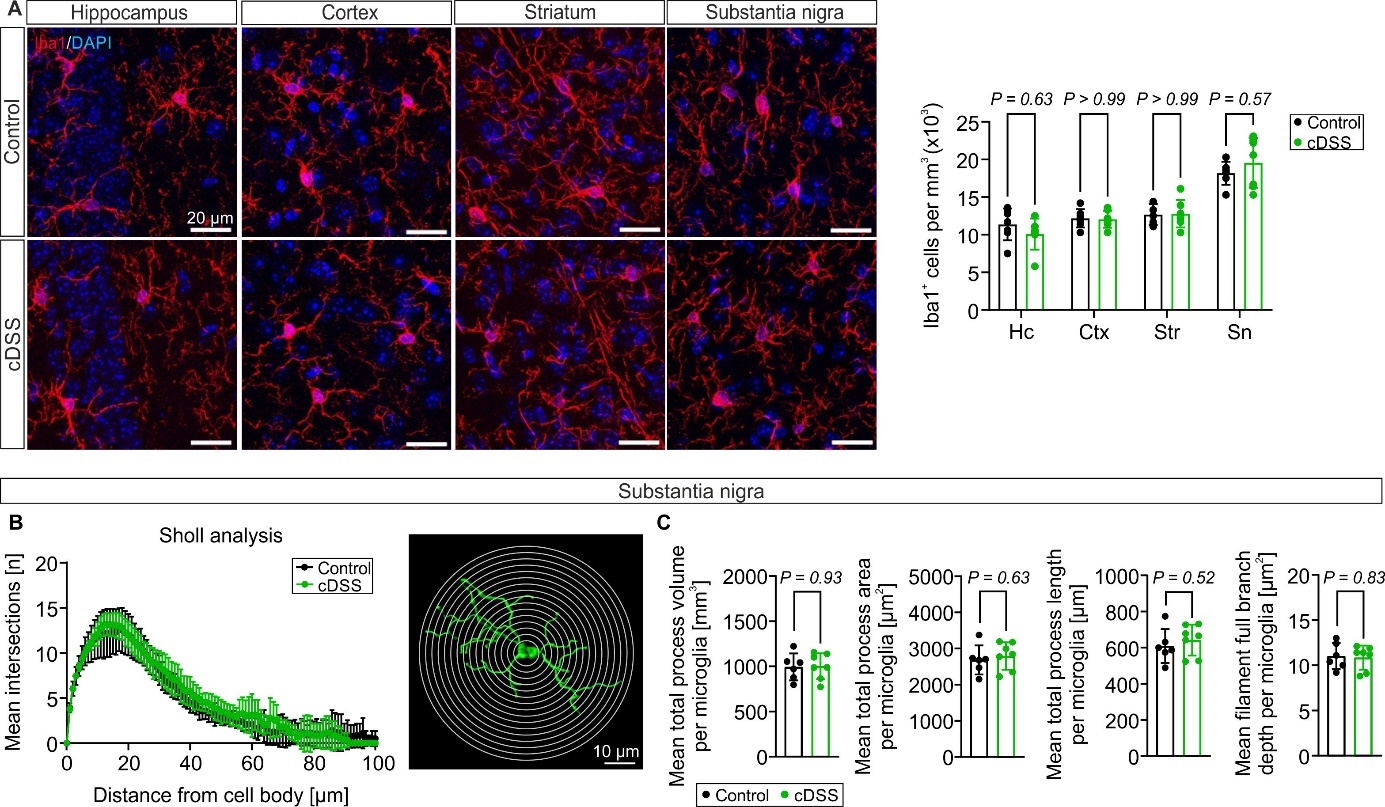


**Figure S4. Regional microglia density and morphology remain unaltered in chronic DSS colitis mice.**

**A** Immunostaining for Iba1 in brain tissue of C57BL/6J mice (left) and quantification of Iba1^+^ cells normalized to mm^3^ in the hippocampus (Hc), cortex (Ctx), striatum (str), and substantia nigra (Sn, *n* = 7 mice per group; Control/DSS: 4 male, 3 female; two-way ANOVA with Šídák's multiple comparisons test). Scale bars, 20 µm. **B** Sholl analysis of microglia in the substantia nigra. **C** Microglial process parameters in the substantia nigra (*n* = 6-7 mice per group; Control: 4 male, 2 female, DSS: 4 male, 3 female; two-tailed, unpaired *t*-test). Each point represents the mean value of one mouse. Data are presented as mean ± s.d.


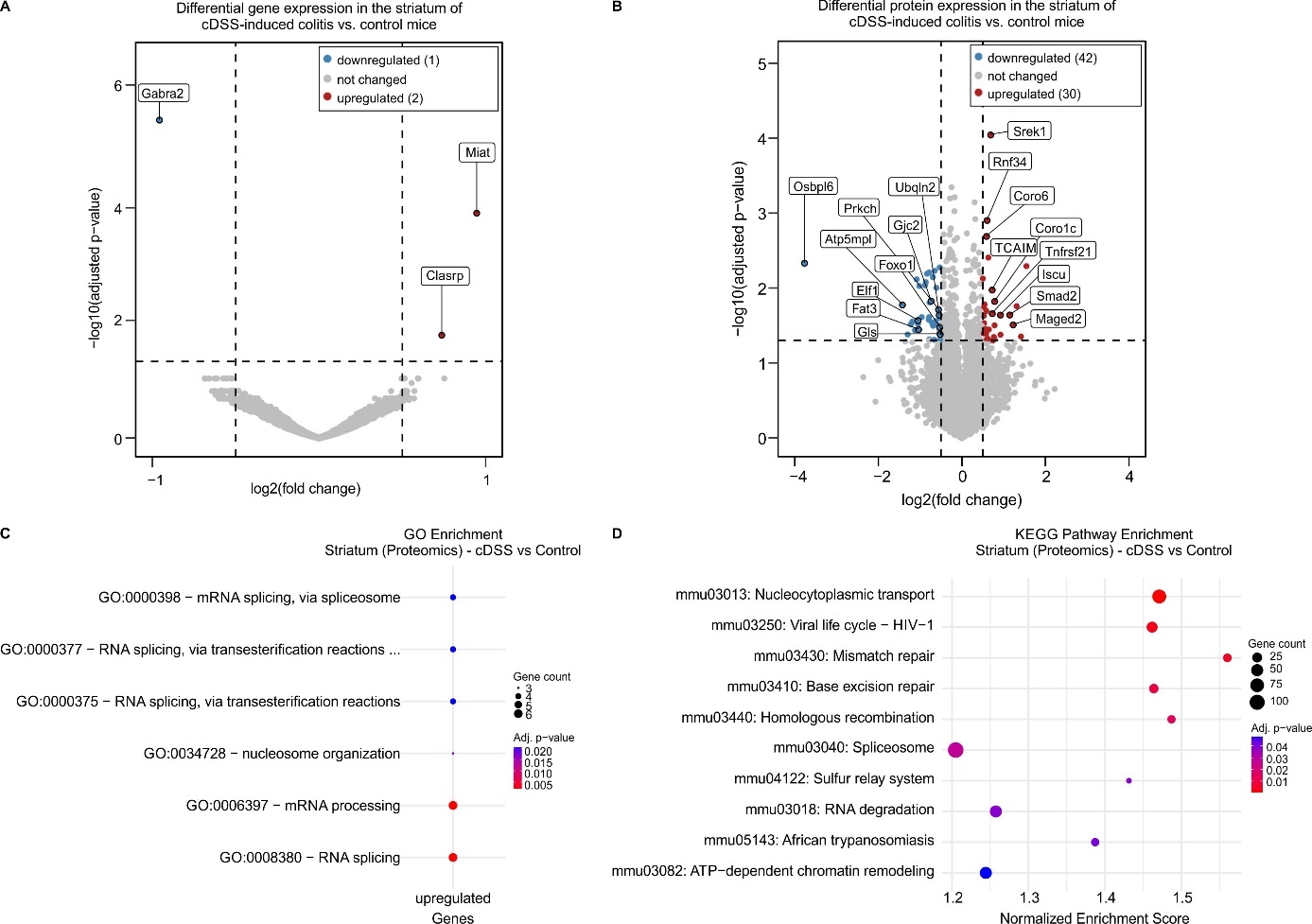


**Figure S5. Transcriptome and proteome analyses reveal subtle alterations in the striatum during chronic DSS colitis. A** Volcano plot of genes differentially expressed in the striatum of chronic DSS colitis mice vs. control mice. **B** Volcano plot of proteins differentially expressed in the striatum of chronic DSS colitis mice vs. control mice. **C** Gene Ontology Biological Process analysis of the 30 proteins significantly higher expressed in the striatum of chronic DSS colitis mice compared to control mice. **D** KEGG (GSEA) pathway enrichment of proteins differentially expressed in the striatum of chronic DSS colitis mice compared to control mice.


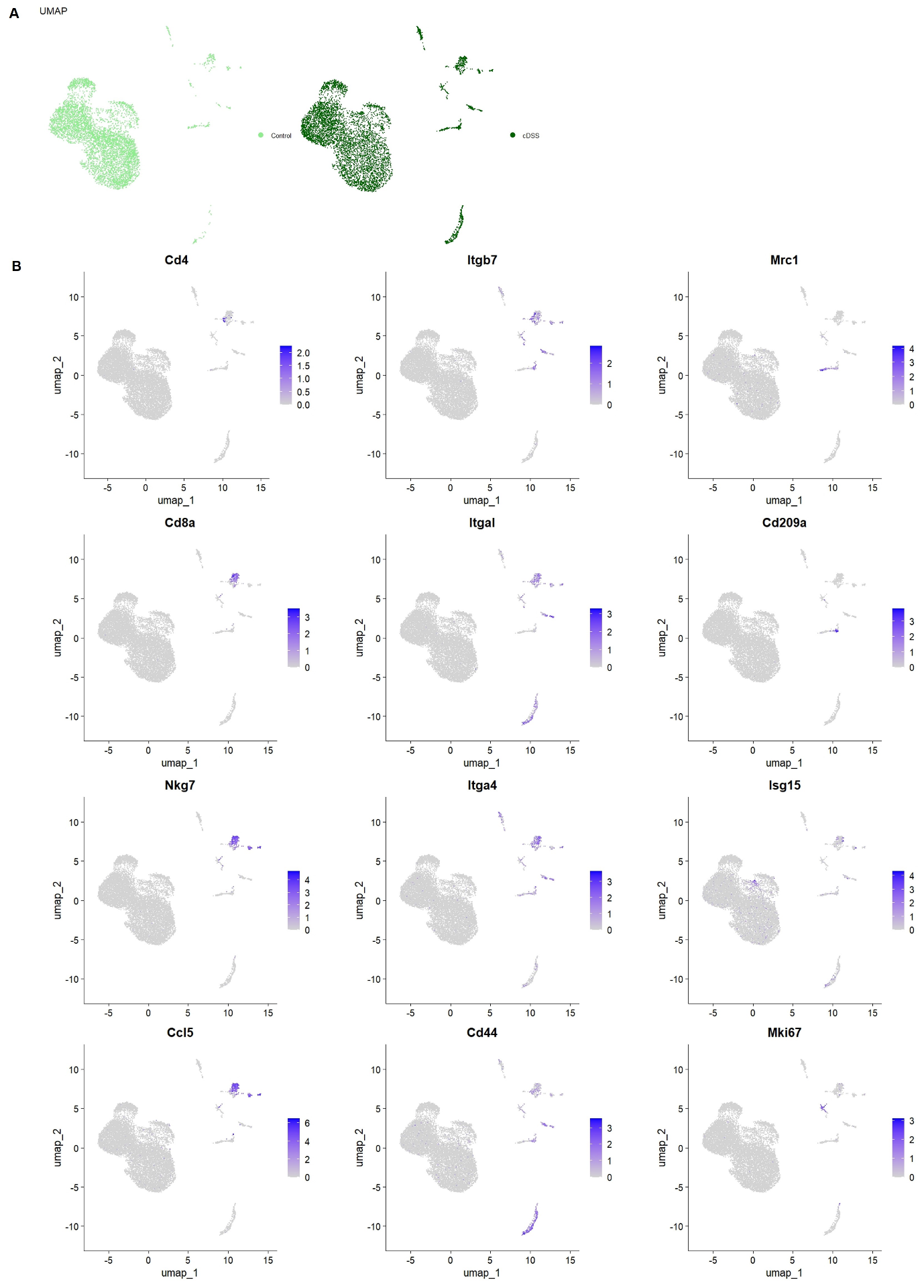


**Figure S6. UMAPs and feature plots of immune cell type defining genes in the midbrain. A** Individual UMAPs of immune cells in the midbrain per group. **B** Feature plots of cluster defining markers.

**Table S1.** **Antibodies used for immunofluorescence and flow cytometry.**

| Antibody | Source | Identifier |
| --- | --- | --- |
| Iba1 polyclonal antibody | Abcam | Cat# ab5076 |
| Iba1 polyclonal antibody | FUJIFILM Wako Pure Chemical Corporation | Cat# 019-19741 |
| C1q monoclonal antibody [4.8] | Abcam | Cat# ab182451 |
| CD4 monoclonal antibody [EPR19514] | Abcam | Cat# ab183685 |
| CD8 monoclonal antibody [EPR21769] | Abcam | Cat# ab217344 |
| CD68 monoclonal antibody [Fa-11] | Bio-Rad Laboratories | Cat# MCA1957 |
| Collagen IV polyclonal antibody | Sigma-Aldrich | Cat# AB769 |
| Ly6G monoclonal antibody [1A8] | BioLegend | Cat# 127602 |
| Phospho-alpha-Synuclein (Ser129) monoclonal antibody [D1R1R] | Cell Signaling Technology | Cat# 23706 |
| TH polyclonal antibody | Sigma-Aldrich | Cat# AB152 |
| TH polyclonal antibody | Thermo Fisher Scientific | Cat# MA1-24654 |
| Donkey anti-Mouse IgG (H+L) High Cross-Adsorbed Secondary Antibody, Alexa Fluor™ 568 | Thermo Fisher Scientific | Cat# A10037 |
| Donkey anti-Goat IgG (H+L) High Cross-Adsorbed Secondary Antibody, Alexa Fluor™ 488 | Thermo Fisher Scientific | Cat# A11055 |
| Donkey anti-Goat IgG (H+L) High Cross-Adsorbed Secondary Antibody, Alexa Fluor™ 568 | Thermo Fisher Scientific | Cat# A11057 |
| Donkey anti-Rabbit IgG (H+L) High Cross-Adsorbed Secondary Antibody, Alexa Fluor™ 568 | Thermo Fisher Scientific | Cat# A10042 |
| Donkey anti-Rabbit IgG (H+L), Alexa Fluor™ 647 | Dianova | Cat# 711-605-152 |
| Donkey anti-Rat IgG (H+L) High Cross-Adsorbed Secondary Antibody, Alexa Fluor™ 488 | Thermo Fisher Scientific | Cat# A21208 |
| Donkey anti-Rat IgG (H+L), Alexa Fluor™ 647 | Dianova | Cat# 712-605-153 |
| eFluor™ 450 anti-mouse CD3e monoclonal antibody [145-2C11] | Thermo Fisher Scientific | Cat# 48-0031-82 |
| APC/Fire™ 810 anti-mouse CD4 monoclonal antibody [GK1.5] | BioLegend | Cat# 100480 |
| Brilliant Violet 650™ anti-mouse CD8 monoclonal antibody [53-6.7] | BioLegend | Cat# 128049 |
| PE/Cyanine7 anti-mouse/human CD11b monoclonal antibody [M1/70] | BioLegend | Cat# 101216 |
| Alexa Fluor™ 660 anti-mouse monoclonal antibody CD19 [1D3] | Thermo Fisher Scientific | Cat# 606-0193-82 |
| PE-Cyanine5.5 anti-mouse CD45 monoclonal antibody [30-F11] | Thermo Fisher Scientific | Cat# 35-0451-82 |
| APC anti-mouse CD45 monoclonal antibody [30-F11] | BioLegend | Cat# 103112 |
| Brilliant Violet 570™ anti-mouse Ly-6C monoclonal antibody [HK1.4] | BioLegend | Cat# 128030 |
| APC/Cyanine7 anti-mouse Ly-6G monoclonal antibody [1A8] | BioLegend | Cat# 127624 |

**Table S2.** **Primer sequences for reverse transcription-quantitative PCR.**

| Gene | Primer forward (5’ - 3’) | Primer reverse (5’ - 3’) |
| --- | --- | --- |
| *Cxcl13* | CTCCAGGCCACGGTATTCTG | CCAGGGGGCGTAACTTGAAT |
| *Hprt* | GTCATGTCGACCCTCAGTCC | GCAAGTCTTTCAGTCCTGTCC |
| *Il1b* | GCAACTGTTCCTGAACTCAACT | ATCTTTTGGGGTCCGTCAACT |
| *Pgk1* | GTCGTGATGAGGGTGGACTT | AACGGACTTGGCTCCATTGT |
| *Tnf* | TAGCCCACGTCGTAGCAAAC | GCAGCCTTGTCCCTTGAAGA |
